# ﻿Species descriptions in myxomycetes – can we settle on rules for good taxonomic practice?

**DOI:** 10.3897/imafungus.16.141199

**Published:** 2025-02-17

**Authors:** Martin Schnittler, Dmytro Leontyev, Iryna Yatsiuk, Anna Ronikier

**Affiliations:** 1 Institute of Botany and Landscape Ecology, University of Greifswald, Greifswald, Germany University of Greifswald Greifswald Germany; 2 Department of Botany, H.S. Skovoroda Kharkiv National Pedagogical University, Kharkiv, Ukraine H.S. Skovoroda Kharkiv National Pedagogical University Kharkiv Ukraine; 3 Institute of Ecology & Earth Sciences, University of Tartu, Vanemuise 46, EE-51014 Tartu, Estonia University of Tartu Tartu Estonia; 4 W. Szafer Institute of Botany, Polish Academy of Sciences, Lubicz 46, 31-512 Kraków, Poland W. Szafer Institute of Botany, Polish Academy of Sciences Kraków Poland

**Keywords:** 18S rDNA, delimitation of species, Eumycetozoa, International Code of Nomenclature for algae, fungi, and plants, molecular barcoding, Myxogastria, taxonomy

## Abstract

*Myxomycetes* are a unique branch of life, recognisable by sporophores showing a fungus-like dispersal biology. These structures bear nearly all diagnostic characters for species identification and develop by rapid transformation of plasmodia. During this short period of time, external factors can significantly influence the formation of morphological characters. Therefore, the description of a new species must be carried out with utmost care. Over the last 50 years, approximately 10–15 new species of myxomycetes have been described per year and only some of the latest publications underpin this with molecular data. In this paper, we discuss a set of recommendations for the description of myxomycete species new to science, striving for the following goals: (i) to minimise the number of erroneous descriptions of the species, whose names later have to be put into synonymy; (ii) to make all respective data easily accessible for the scientific community; and (iii) to comply with existing rules of nomenclature. We recommend (1) whenever possible not to describe a new taxon from a single specimen; however, an exception could be made only if supported by molecular data and by unique morphological characters which are unlikely to fall in the range of infraspecific variation of related species; (2) preparing detailed descriptions, including data on developmental stages, microhabitats, ecology, phenology and associated species; (3) providing at least two independent diagnostic characters that tell the new species apart from all others; (4) obtaining a molecular barcode and, whenever possible, providing proof for reproductive isolation of the new species from related taxa; and (5) depositing type specimens in public herbaria. To comply with nomenclatural rules, (6) the new name must be registered in a recognised repository, (7) all published names should be checked for usability before proposing a new name and (8) a unique name should be chosen, preferably highlighting a distinct character of the new species.

## ﻿Introduction

Recent advances in molecular barcoding methods, as well as in photographic techniques, which now allow the direct acquisition of high-resolution images in the field, have led to a renewed interest in myxomycete taxonomy. This has resulted in a growing number of non-institutional researchers who are discovering new species (e.g. Lloyd et al. (2024b); Gøtzsche et al. (2025)) and this is what we need to explore the real diversity of myxomycetes on this planet.

This fortunate trend also bears two challenges. First, we must find ways to describe new species in a fully comprehensive manner, ensuring that the entire research community can instantly consider these descriptions for further work. Second, we must ensure that both phenotypic plasticity and intraspecific variation will not erroneously cause descriptions of new species. If these two conditions are not fulfilled, premature descriptions arise, creating information noise and causing a burden for future taxonomic research (Schnittler and Mitchell 2000). Proving the synonymy of such names with an existing species is laborious and funding for such research is difficult to find.

What makes myxomycetes special in comparison to many other groups of organisms when it comes to the description of species new to science? Several features can be invoked:

1. In contrast to multicellular organisms, myxomycetes display only a limited set of morphological traits and nearly all of these traits are linked to the sporophore as the site of the production of spores (Clark and Haskins 2014). As a result of frequent convergent evolution, non-related species appear quite similar in their morphology (as shown for *Physarales*, García-Martín et al. (2023)). Some taxa, such as *Licea* spp., display trends towards reductive evolution; many *Perichaena* spp. and *Didymium* spp. lack a stalk and a capillitium and, thus, a whole corresponding set of characters. We therefore need to explore as many accessible morphological features as possible.
2. Myxomycete sporophores develop not through a growth process, but through the reformation of the existing biomass of a plasmodium within a short time, usually hours to days. Weather events during that period can greatly alter morphological characters and increase phenotypic plasticity. This was shown for *Lepidodermacrassipes* (Flatau et al. 1987) and *Lepidodermastipitatum* (Flatau 1984), which were reported to be distinguishable by the pattern of lime scales covering the surface of fruiting bodies. Molecular phylogenies showed the first taxon to be synonymous with *Didermatigrinum* (formerly *Lepidodermatigrinum*), while the second turned out to be a synonym of *Didermafloriforme* (Ronikier et al. 2022). The latter authors showed the transition from dense and small lime scales (the diagnostic character of *L.crassipes*) to scattered and large scales (in *D.tigrinum*) within a single colony stretching over a few centimetres. We must conclude that changes in environmental conditions, especially moisture, at: (i) short times and/or (ii) within cm ranges may cause differences in the formation of lime scales – structures traditionally given high taxonomic value for myxomycetes (Martin and Alexopoulos 1969).

Seemingly eye-catching morphological characters may be misleading: in the type material of *Arcyriapapilla*, the upper part of the sporotheca forms a “nipple-like structure” and this was given as a diagnostic feature of the new species (Ejale and Gill 1992). This is likely an abnormality that often occurs if a developing sporosphore dries out too quickly. A molecular investigation of the type material (deposited at the University of Benin and the Commonwealth Mycological Institute, London) may reveal that the taxon is identical to another species of the genus *Arcyria*. Until such an investigation is carried out (given the conservation status of the material allows), the taxon name remains in use (Lado 2005–2024).

Another trait associated with frequent malformation is the capillitium, where granules, nodules, knots or swellings may form, probably from accidentally included material. *Lamprodermagranulosum* (Neubert et al. 1990) was reported to differ from *L.puncticulatum* in the presence of capillitial granules (Schnittler et al. 2010). This characteristic, however, is not linked to a particular genotype (Fiore-Donno et al. 2011); thus, the species must be regarded as synonymous with *L.puncticulatum*. However, further studies, including barcoding of the type specimens, are needed to verify the identity of *L.granulosum*.

Due to the significant phenotypic plasticity of diagnostically important structures, one or two specimens of myxomycetes never adequately reflect the range of intraspecific variation. For instance, *Alwisiarepens* was described from two gatherings encountered in the same area. Both specimens displayed a creeping stalk and this character was considered in the description of the species and even predetermined the choice of its epithet (Leontyev et al. 2014). However, further gatherings had erect stalks and revealed several characters not observed in the original material, such as a dehiscent peridium and a rudimentary capillitium (Lloyd et al. 2024a).

3. (Many species of myxomycetes can be observed in moist chamber cultures (Gilbert and Martin 1933). For species with minute sporophores, like *Echinosteliumbisporum* (Schnittler et al. 2015) or those bound to special substrates, like the fimicolous myxomycetes (Eliasson and Keller 1999), this may be the only reliable way to detect sporophores. Therefore, we have cases of species descriptions based exclusively on specimens obtained from moist chamber cultures (e.g. Stephenson et al. (2018); Sadykov and Potapov (2023); Vlasenko and Vlasenko (2023)). However, the constantly humid conditions in these cultures may cause aberrant and malformed sporophores, especially in lime-containing taxa.
4. Case studies conducted within the last decade revealed that several species exhibit a pronounced internal genetic structure. We frequently detect reproductively isolated groups with independently inherited genetic markers, which constitute putative biospecies. Examples are *Trichiavaria* (Feng and Schnittler 2015, three recombination groups for three markers and group I introns), *Hemitrichiaserpula* (Dagamac et al. 2017, four groups for two markers, partially with differences in spore ornamentation), the species complex around *Badhamiaalbescens* (Shchepin et al. 2022, > 15 groups for three markers) or *Diacheopsisresinae* (Gøtzsche et al. 2025, three groups for two markers and alleles of the nuc EF1A gene). Without studying the internal genetic structure and trait variation in such complexes, a correct description of these groups at the species level will not be possible.

With all these factors taken into account, the description of new species of myxomycetes may be more challenging than for other groups of terrestrial macro-organisms. In this paper, we: (1) provide some basic data on descriptions of new species of myxomycetes from Linnaeus to the present day and (2) offer a list of good practice recommendations, the implementation of which, in our opinion, will help improve the quality of descriptions of new species and minimise possible errors.

## ﻿Materials and methods

The recommendations written here were discussed within the framework of the 11^th^ International Congress on Systematics and Ecology of Myxomycete (ICSEM11), held in Tartu, Estonia, on 28–31 August 2023 and, in addition to the contributions from the authors, numerous suggestions from the audience were included in this paper.

Data for the description of taxa per year were taken from the Catalogue of Life (https://www.catalogoflife.org/data/, accessed 18.07.2023); in decisions about acceptance of names, this database mainly follows the nomenclatural database (Lado 2005–2024). It currently includes 2670 validly described names and new combinations. To monitor the increase in taxonomic activity over time, we counted: (1) the total number of validly published species descriptions, (2) the number of currently accepted basionyms (the species names that were never recombined), (3) the number of validly published new combinations and (4) the number of currently accepted new combinations for each year. Adding numbers for (2) and (4) yields the number of accepted taxa (5) and the remaining names are those currently regarded as synonyms (6).

All names of species cited as examples can be found in the nomenclatural database (Lado 2005–2024).

## ﻿Results

### ﻿Species descriptions over time

**Figure 1.**
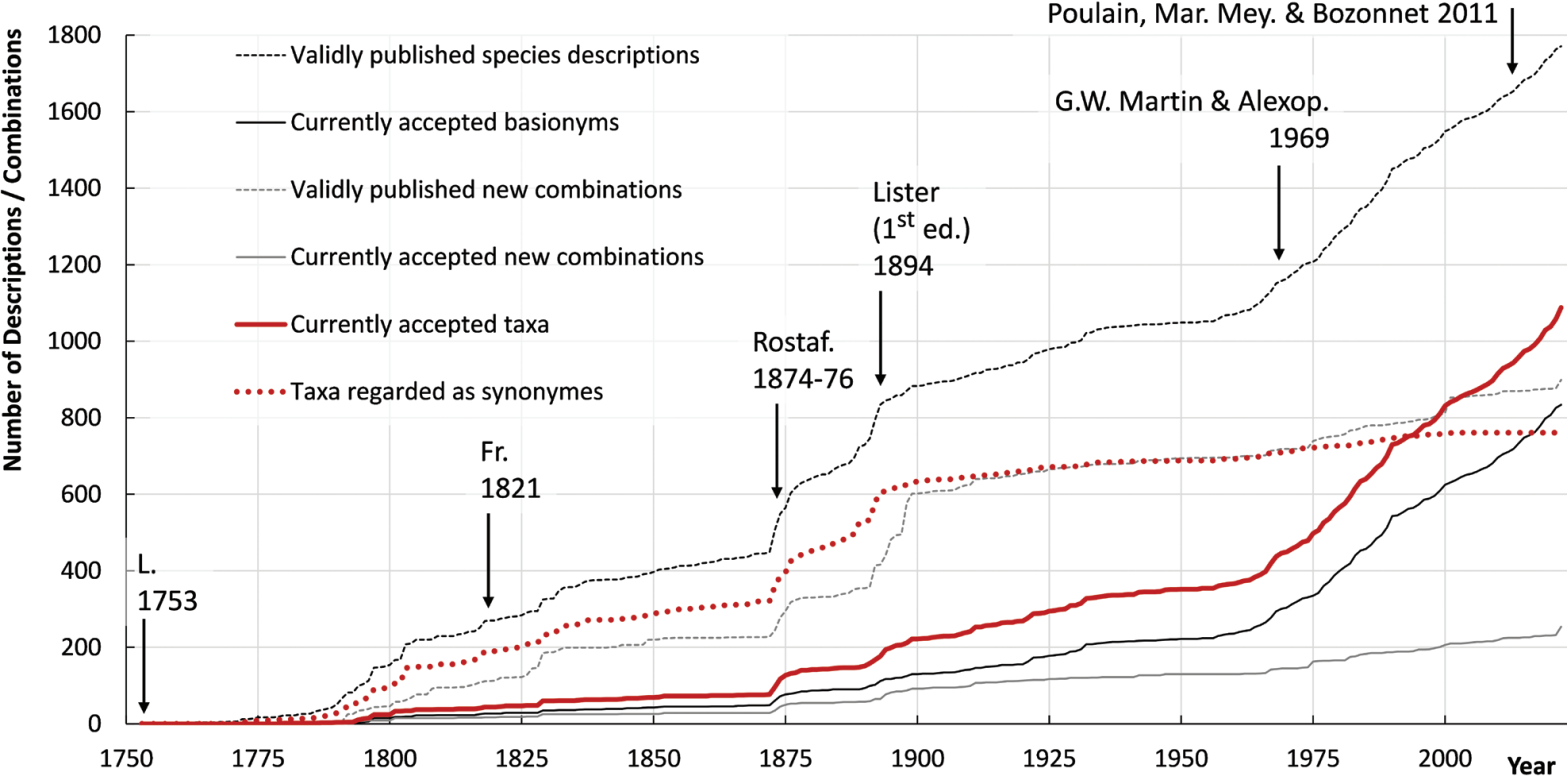
Descriptions of new taxa and new combinations (at species rank) within the class *Myxomycetes* from 1753 to 2022 according to the Catalogue of Life (release 18.07.2023, https://doi.org/10.48580/dfsy). The database lists 1088 currently accepted taxa and an additional 761 are regarded as heterotypic synonyms. Arrows indicate the publication dates of major monographs (Linné 1753; Fries 1821; Rostafiński 1874, 1875, 1876; Lister 1894, 1911, 1925; Martin and Alexopoulos 1969; Poulain et al. 2011), with the authors given with their abbreviations used in species descriptions.

The number of morphologically distinguished and accepted myxomycete species now approaches 1100 and the trend of increasing numbers of new species described per year (Schnittler and Mitchell 2000) is likely to continue (Fig. 1). The annual rate of new descriptions increased considerably after the publication of the influential monograph of Martin and Alexopoulos (1969) and has since stabilised at a level of ca. 12 species per year. However, over the last 5 years, an increase to > 15 species per year has been observed, reflecting the increasing interest in myxomycetes and advances in techniques used in taxonomy, including molecular barcoding and microphotography. Many of the names of species described between 1875 and 1900 are now regarded as heterotypic synonyms, but virtually all of the species described later are accepted.

### ﻿Good taxonomic practice in myxomycete descriptions

The following recommendations aim: (i) to minimise the number of erroneous descriptions of the species by a comprehensive description including molecular characters and clear diagnostic features, (ii) to make all these data easily accessible for the scientific community and (iii) to comply with existing rules of nomenclature. Our eight recommendations can be divided into three parts: species delimitation (1–4); registration and preservation of types (5); and nomenclatural issues (6–8). The latter essentially require a species description to be published “effectively” (in a way that the scientific community can access it), “legitimately” (under a unique name) and “validly” (with diagnosis, description and reference to a type). A useful reference is the paper of Seifert and Rossman (2010) about description of fungal species (but note that a diagnosis can now as well be done in English and the rules of effective publication of on-line published papers changed).

### ﻿Recommendation 1. Do not describe singletons without molecular support

1.1. A new species should be known from more than one gathering (see ICN, International Code of Nomenclature for algae, fungi and plants; Turland et al. (2018), Art. 8.2. footnote) and from more than one locality. This reduces the risk of describing a taxon based on rare phenotypic aberrations and helps to correctly assess within-species variation in morphological and molecular characters. Special care should be given to taxa known from only moist chamber cultures, which often show aberrant structures. This concerns especially the formation of lime-containing structures, such as peridial scales, crystals or lime nodes.

1.2. Description of the new species from a single gathering can be considered only as an exception. If a singleton specimen displays at least two striking morphological characters which are likely to be outside the intraspecific variation of the respective characters in related species and its barcode is unique and outside the 99.1% similarity range found empirically to be applicable for molecular species differentiation (Borg-Dahl et al. 2018, but see as well Yatsiuk et al. (2023) and Inoue et al. (2024) for application), the description of a new species may be considered. Examples are the recent descriptions of *Trichiatuberculata* (Leontyev et al. 2024) based on a single gathering and *Spiromyxaslocanensis* (Yatsiuk et al. 2024) based on two gatherings made at different times from the same locality.

1.3. To facilitate the search for more material, morphological characters of single specimen that may be candidates for a new taxon can be described and illustrated in a specialised journal such as Slime Molds (https://www.slimemolds.org) using a preliminary name in English or Latin which best highlights a prominent character of the putative new species. Preliminary names should be accompanied by the additions *nom. prov.* (*nomen provisorium*, provisional name) or *ad int.* (*ad interim*, temporarily). Images can also be posted in internet forums, such as the “Slime Mold Identification & Appreciation” group on Facebook (https://www.facebook.com/groups/1510123272580859) to increase attention, which can lead to further observations. Social media are not stable data repositories, but they represent an efficient public platform for the information exchange. When additional material from different locations becomes available, a new species can be formally described in a scientific journal.

### ﻿Recommendation 2. Provide a detailed description

2.1. A new taxon should be described as comprehensively as possible (see ICN, Recommendation 39A). This includes all fruiting body structures, but as well plasmodium appearance and developmental stages, if observed. Whenever possible, images in the field should be taken to document the immature stages since the colour of immature sporophores can, in some cases, be quite helpful (as in *Tubifera* spp. and *Lycogala* spp., see Leontyev et al. (2015, 2023b)). Images from both the dissecting microscope and compound microscope should be provided. Try as well to obtain SEM micrographs of all relevant structures (capillitium, inner and outer side of the peridium, spores); here, cooperation between institutional and non-institutional researchers will help. Illustrate all diagnostic characteristics of the new species (see ICN, Recommendation 38D).

2.2. Spore diameter should be measured for at least 30 spores (Parmasto and Parmasto 1987); obviously oversized or mal-developed spores should be excluded. If spore size is assumed to be a decisive character for the erection of a new species, attempt to measure even more spores in several specimens (see Woyzichovski et al. (2022) for an automated approach for spore measurement). We suggest providing the raw data for measurements as supplementary material to the published article or as supplements available through open-access platforms.

2.3. Collect data on geography, ecology, phenology and associations with other organisms. Provide exact geographic references and coordinates for all specimens of the new taxon. Take photos of the micro-habitat of the new species and describe it as exactly as possible (more than just “decaying wood”). Note steady associations with other organisms that can be indicator species for a given taxon. For example, the liverwort *Nowelliacurvifolia* can serve as an indicator species for the minute myxomycete *Barbeyellaminutissima* (Schnittler et al. 2000). As shown for the recently described *Diacheopsisresinae* (Gøtzsche et al. 2025), a micro-habitat (the substrate plus microclimate suitable for a species) can be distinctive and may be inhabited by a specific community of microorganisms, which can be characterised by metabarcoding.

2.4. Make all primary data available and easy to re-use. Publish photographs of all examined specimens and localities, tables with measurements, sequences, alignments, trees etc., as supplementary materials to the paper and/or in public repositories. Try to meet the FAIR principles for data management (Wilkinson et al. 2016).

### ﻿Recommendation 3. Try to identify as many diagnostic characters as possible

3.1. Give a diagnosis as well as a description of the new species. The diagnosis is “a statement of that which in the opinion of its author distinguishes the taxon from other taxa” (ICN, Art. 38.2). According to Hassemer et al. (2020), the diagnosis is a synthetic statement of the morphological characters that allow us to distinguish the new taxon from its relatives and should ideally be as concise as possible. In contrast, the description is an analytical statement describing comprehensively all features of the new taxon, including as well anatomical, biochemical, karyological and molecular aspects, if available. It should ideally be as thorough as possible.

3.2. Try to find traits that tell the new species apart from others and include such descriptive elements in the diagnosis (see ICN, Art. 38). A new taxon should differ in at least two preferably qualitative characters from its most morphologically similar relative. Include in your research: (i) all morphologically most similar described taxa, but as well (ii) taxa with similar molecular barcodes (see below), to provide a convincing list of traits distinguishing the new species from related taxa. Additionally, you can provide an identification key for the genus or the group of species to which the new taxon belongs.

3.3. Special care should be given for differences in quantitative characters. Here, especially broad sampling is needed, including several sporophores from several gatherings. In myxomycetes, differences in spore size of 0.5–1 µm are likely to be within the range of intraspecific variation (as it was shown for species like *Physarumandinum*, Ronikier and Lado 2013; *Badhamiaalbescens*, Woyzichovski et al. 2022; *Diacheopsisresinae*, Gøtzsche et al. 2025).

### ﻿Recommendation 4. Obtain a molecular barcode

4.1. At least one genetic marker should be sequenced for the holotype of a new species and only barcoded specimens should be chosen as holotypes. The current standard for molecular barcoding of myxomycetes is the first part of the 18S rDNA gene (nucSSU, Schnittler et al. (2017); Leontyev and Schnittler (2021), see Fiore-Donno et al. (2008, 2012, 2013) for primers), but other marker genes can also be used. A unique barcode is one of the most convincing arguments that the new taxon deserves species rank. This approach allows the comparison of putatively new species with barcodes of closely-related taxa. Non-institutional researchers should try to link with colleagues from institutions with molecular laboratories – only a few hundred spores are needed to barcode a species and protocols for the procedure are available (Janik et al. 2020b; Schnittler et al. 2020). When submitting to NCBI GenBank, label the sequence with the name of the new species and annotate it is a holotype in the specimen description. This would facilitate further use of the molecular barcode of the type specimen as a reference sequence. Since DNA is often highly fragmented in herbarium specimens older than 15 years and will become increasingly difficult to sequence in old material, it is important to barcode a specimen upon description.

4.2. Check and discuss molecular differences of the new species to the most similar taxa identified by a BLAST search in GenBank (see recommendations by Kirschner et al. (2023) and Yeh et al. (2023)). This helps to find identical sequences obtained within other studies and to recognise sequences accidently obtained from contaminations.

4.3. A new barcode alone, even if it shows a significant difference from the nearest known barcode, does not make a new species. One species can display several genotypes for a given marker gene (Shchepin et al. 2022; Leontyev et al. 2023a). The barcode gap, which represents the difference between inter- and infraspecific variability, is a useful tool for delimiting species (Borg Dahl et al. 2018), but it is not constant across larger groups (Janik et al. 2020a, c; Yatsiuk et al. 2023). For instance, accessions from the species pair *Polyschismiumfallax*/*P.peyerimhoffii* may differ by only three bases in the ca. 500 bp long 18SrDNA barcode (< 1%, Inoue et al. (2024)), whereas the difference within accessions of *Lamprodermascintillans* may exceed 5% (Yatsiuk et al. 2023). Therefore, a new barcode sequence may still be part of the infraspecific variation.

4.4. If the barcode of a new taxon is identical or extremely close to that of an existing taxon, the position of a putative species should be verified by a second, independently inherited marker. Good candidates include the nuclear single-copy genes EF1A (Novozhilov et al. 2013a; Wrigley de Basanta et al. 2017; Ronikier et al. 2020) and α-Tub (García-Martín et al. 2023) and the mitochondrial multicopy genes COI (Feng and Schnittler 2015) and mtSSU (Lado et al. 2022; García-Martín et al. 2023). The last gene seems to be most easily obtainable using nearly universal primers (García-Cunchillos et al. 2022, but *Meriderma* spp. do not fit entirely). However, it shows on average less variation than nucSSU.

4.5. Amoebozoa in general and *Myxomycetes* in particular are sexual organisms (Lahr et al. 2011; Spiegel 2011; Hofstatter et al. 2018) and a biological species concept fits well with this group. Thus, if you are able to obtain an independently inherited second genetic marker, you may use it for a molecular test of reproductive isolation (see Feng and Schnittler (2015); Shchepin et al. (2022); Leontyev et al. (2023a)). This will show whether the new taxon complies with the biological species concept and helps to delimit it more precisely.

4.6. With the current level of knowledge in myxomycete genetics, but as well existing limitations in barcoding technology, at present, we do not recommend for myxomycetes a formal description of cryptic species. While these taxa comply with the biological species concept by being reproductively isolated, no distinguishing morphological traits have been found so far. A well-investigated example concerns three biological species within *Trichiavaria* (Feng and Schnittler 2015).

4.7. It is highly recommended to build a phylogeny that includes all available barcodes for the new species, but also the broadest possible sampling of DNA sequences of related taxa retrieved from GenBank. The phylogeny may help to test whether the new taxon: (i) represents a monophyletic group and (ii) does not branch within any other taxon. However, short 18S rDNA barcode sequences might not always be sufficient to prove the monophyly of a new taxon by obtaining a high statistical support for the tree nodes; in this case, it is useful to investigate at least two independent markers.

### ﻿Recommendation 5. Deposit vouchers in public herbaria

5.1. Clearly designate type specimens according to the rules of the ICN. Select a holotype and, if possible, isotypes. Deposit type specimens in one or several public herbaria and publish the obtained accession numbers together with your personal collection number. When you plan to deposit isotypes in other herbaria, make sure that the personal collection number has been incorporated into the records of this collection. Since a species name is permanently attached to the type specimen (ICN Art. 7.2, see also Recommendations 7A, 9C, 40A.6), the type specimens remain the first source for all further taxonomic studies (Ronikier et al. 2022). The availability of type specimens greatly facilitates further research.

5.2. Spores taken from the holotype may be stored in plastic tubes separately from the specimens at 4 °C for further molecular studies; data from ferns suggest this treatment to conserve nuclei and thus DNA for future molecular studies (Tang et al. 2023).

5.3. Permanent microscope slides can be prepared using a stable mounting medium. We recommend the use of Hoyer’s medium (Anderson 1954) as a standard, noting that measurements of microscopic structures can differ when using different media (Booth et al. 1971; Alexopoulos et al. 1996). The preparation of permanent microscope slides reduces repetitive destructive sampling from type specimens for microscopic studies. Always mention the medium used to obtain measurements for quantitative characters.

### ﻿Recommendation 6. Register a name in a recognised repository

6.1. According to nomenclature, myxomycetes are treated as fungi and, thus, fall under the provisions of Chapter F of the ICN (May et al. 2019). Since 1 January 2013, to be validly published, a name must have an identifier issued by one of the three recognised repositories (Fungal Names, Index Fungorum, MycoBank; see Redhead and Norvell (2013) and May et al. (2019)). For myxomycetes, MycoBank is most commonly used. Registration in one repository is mandatory and ensures that a new name will not be overlooked.

### ﻿Recommendation 7. Check for synonyms published earlier

7.1. If a new species is split off from an existing one, all synonyms must be considered as candidate names for the new taxon. Studies of the respective type specimens may reveal that one or more earlier proposed names (later regarded as synonyms) belong to the new taxon. In this case, the earliest name should be used for the taxon you are planning to describe.

### ﻿Recommendation 8. Choose the species name carefully and publish it properly

8.1. Avoid choosing an epithet already in use in any related myxomycete species (see ICN, Recommendation 23A.3(h)). Epithets already in use may become homonyms if future investigations place two species from different genera into a single one. In such cases, the semantic link between the new and the old name can be lost. In *Mucilagocrustacea*, molecular data suggested its inclusion in the genus *Didymium*, but here, the species *Didymiumcrustaceum* already exists (Lado 2005–2024); thus, a new epithet was needed. Choosing the generic name “*Mucilago*” as an epithet to keep the link produced an illegitimate name (Prikhodko et al. 2023), since, in such a case, the earliest available legitimate name of the species must be chosen (see ICN, Art. 11.4). Thus, the new legitimate name is *Didymiumspongiosum* (García-Martín et al. 2023), taken from the basionym *Mucorspongiosus* published in 1783 (Lado 2005–2024).

8.2. Avoid choosing a genus name already in use in any other group of organisms, including those that fall under the regulations of the International Code of Zoological Nomenclature (ICZN). *Myxomycetes*, along with dictyostelids, have always fallen under the provisions of the Botanical Code (ICN, Pre. 8), although other groups of *Amoebozoa* are governed by the Zoological Code (ICZN). Ronikier and Halamski (2018) showed that a change to the latter code, as seems logical from phylogenetic data, would cause severe problems due to several generic names already in use for animal groups (e.g. *Trichia*, a genus of crabs). Since a future decision to change the code cannot be ruled out, one should not enlarge the problem by choosing generic names that are already in use. For names published after 1 January 2019, when the name of a myxomycete genus or species is identical to a prokaryotic or protozoan name, it is illegitimate (ICN, Art. F.6.1; May et al. (2019)).

8.3. Follow the rules and recommendations of ICN (Art. 23, Art. 60) when creating a new epithet. Although the species epithet may be taken from any source (ICN, Art. 23.2), we recommend avoiding non-informative epithets, including personal and geographic names (see ICN, Recommendation 23A.3(j)), unless the name denotes especially high abundance in some regions or proven endemism (such as the genus *Tasmaniomyxa* which is very likely endemic to the temperate zones around the Tasman Sea, Lloyd et al. (2024b)). Whenever possible, use epithets describing a macro- or micromorphological character distinguishing the new species from others. Such a practice facilitates quick recognition of a new species.

## ﻿Discussion

### ﻿Accumulation of data about myxomycete diversity

Together with the *Arcellinida* (testate amoebae), the myxomycetes are one of the few groups of protists where a morphological species concept is readily applicable. The obvious reason is the sporophore (Schnittler et al. 2012), which can be stored in herbaria, such as plants and fungi. The first illustration of a myxomycete species was probably *Lycogalaepidendrum*, given by Pankovius (1654). However, the nomenclatural starting point for the group, along with fungi and most plants, is Linné’s species Plantarum (1753) (Art. F.1.1, May et al. (2019)). Until de Bary (1864) recognised slime moulds as protists, their sporophores were described as a kind of little puffballs and, indeed, some genera, such as *Lycogala*, show dispersal adaptations that are very similar to those of puffballs (see Leontyev et al. (2022)).

Rostafiński’s (1874, 1875, 1876) monograph marked the first systematic treatment of the group. Increasing collecting activities in the “golden age of botany” between 1850 and 1900 caused a rapid increase in species descriptions, but many proposed names were later put to synonymy (Fig. 1). The first consolidation came with Lister’s monographs (1894, 1911, 1925). The 20^th^ century saw a slow, but steady increase in species description until the influential monograph of Martin and Alexopoulos (1969). Since then, the annual rate of new descriptions has increased considerably. This may be caused by the application of scanning electron microscopy (SEM) in myxomycete taxonomy, which allowed us to document spore, capillitium and peridium ornamentations in unprecedented detail. The current rate of ca. 15 new descriptions per year is likely to increase, especially with the accessibility of good microscopic and photographic equipment to non-institutional researchers.

### ﻿Molecular barcoding in myxomycete taxonomy

Only a decade ago, with two papers outlining the molecular systematics of myxomycetes (Fiore-Donno et al. 2012, 2013), molecular investigations became a standard, first for phylogenetic investigations and, with some delay, for the description of new species. Barcoding, as a tool for species identification and delimitation, was introduced to myxomycete studies around 2013 (e.g. Novozhilov et al. (2013b); Leontyev et al. (2014)) and relies predominantly, as in most other protist groups (Adl et al. 2014), on the first part of the ribosomal RNA operon (18S rDNA, see Schnittler et al. (2017)). Later, several more marker genes inherited independently from 18S rDNA were introduced to produce more robust phylogenies (Dagamac et al. 2017; Leontyev et al. 2019; García-Martín et al. 2023; Prikhodko et al. 2023). However, even today, many species descriptions are not accompanied by barcodes and we still lack a systematic project for barcoding all known taxa of myxomycetes. Such a project, if initiated, would likely significantly increase the number of described myxomycete taxa by discovering unrecognised or even morphologically unrecognisable (cryptic) species. Simultaneously, molecular barcoding may reveal that some previously described species are synonyms.

### ﻿Good taxonomic practice

The recommendations in this paper, which specify those of the ICN (Turland et al. 2018; May et al. 2019), are not seen by us as strict rules. Research work is unpredictable (and this is one of its main challenges) and taxonomic decisions cannot be dogmatised. These recommendations are intended to be a guideline for well-prepared species descriptions to make them more convincing, comprehensible and usable for further research, particularly for the major treatments in molecular phylogeny. We hope that this paper will encourage institutional researchers to provide services to barcode specimens of candidate species found by non-institutional colleagues to verify future descriptions. Introducing new genera of myxomycetes would require other recommendations, but since such guidelines for new genera of fungi have been proposed by mycologists (Vellinga et al. 2015), we strongly recommend following these guidelines when new genera of myxomycetes are considered.

## ﻿Additional information

### Adherence to national and international regulations

We here adhere to the ICN, International Code of Nomenclature for algae, fungi and plants; Turland et al. (2018).

### Author ORCIDs

Martin Schnittler https://orcid.org/0000-0003-0909-5627

Dmytro Leontyev https://orcid.org/0000-0002-4122-1091

Iryna Yatsiuk https://orcid.org/0000-0003-1610-3533

Anna Ronikier https://orcid.org/0000-0003-0193-8685

### Data availability

The dataset analysed during the current study is available in the Catalogue of Life (release 18.07.2023, https://doi.org/10.48580/dfsy).
